# The Munich Wrist Questionnaire (MWQ) – development and validation of a new patient-reported outcome measurement tool for wrist disorders

**DOI:** 10.1186/s12891-016-1029-4

**Published:** 2016-04-14

**Authors:** Marc Beirer, Julian Serly, Helen Vester, Dominik Pförringer, Moritz Crönlein, Stephan Deiler, Peter Biberthaler, Chlodwig Kirchhoff

**Affiliations:** Department of Trauma Surgery, Klinikum rechts der Isar, Technical University of Munich, Ismaningerstrasse 22, Munich, 81675 Germany

**Keywords:** MWQ, Outcome measurement tool, Validity, Reliability, Responsiveness

## Abstract

**Background:**

Although self-assessment questionnaires for the wrist joint are numerous, most validation studies focus on a specific pathology and patient collectives. In addition the available questionnaires focus on subjective parameters such as pain, usual and specific activities but the range of motion (ROM) as an essential objective parameter in wrist disorders is rarely considered. Therefore the purpose of the presented study was to develop and validate a new universally applicable self-assessment score, the Munich Wrist Questionnaire (MWQ), which allows for the assessment of subjective as well as objective parameters of the wrist joint.

**Methods:**

The MWQ consists of 16 items addressing three domains: pain, work and activities of daily living and wrist function including range of motion and grip strength. In a prospective clinical study validity, reliability and responsiveness of the MWQ of physical active patients were evaluated.

**Results:**

Validation study included 100 patients (mean age 41 years, SD 16.3 years; range, 18–77 years). Test-retest reliability was substantial, with intraclass correlation coefficients ranging from 0.75 to 0.83 for the three domains. Construct validity and responsiveness were confirmed by correlation coefficients of at least 0.86 for construct validity and for responsiveness ranging from 0.61 to 0.65.

**Conclusions:**

The MWQ presents a valid and reliable instrument for a qualitative self-assessment of subjective and objective parameters (e.g. range of motion) of the wrist joint. Quantitative measurement of wrist function may not longer be limited to specific wrist disorders or patient groups. The MWQ seems to allow for a broad application in clinical research and may facilitate the comparison of treatment results in wrist disorders.

**Electronic supplementary material:**

The online version of this article (doi:10.1186/s12891-016-1029-4) contains supplementary material, which is available to authorized users.

## Background

Clinical scoring systems became more and more popular in evaluating the efficacy of treatment procedures in wrist disorders [1, 2]. Numerous physician-based as well as patient-reported clinical measurement tools have been developed. However the physician-based clinical examination does not necessarily correlate with the patient’s satisfaction [3] and does not inevitably take into account further aspects related to an analysis of outcome such as the patient’s ability to perform activities of daily living and the ability to return to previous occupations [1]. Therefore the additional use of self-assessment questionnaires to clinical assessed parameters may result in a higher transparency of the patient’s wrist function and restrictions. A systematic review of the literature was performed to identify valid and commonly used scoring systems regarding follow-up examination in the field of wrist disorders. PubMed.gov was searched for wrist-specific terms (wrist, surgery, joint, upper extremity) combined with psychometric (validity, reliability, responsiveness, follow-up) and instrument specific terms (self-evaluation, patient-based, measurement tool, outcome measure, questionnaire). The Disabilities of the Arm, Shoulder and Hand (DASH) [4], the Patient-Rated Wrist Evaluation Score (PRWE) [5], the Cooney and Bussey Score (CBS) [6] and the Mayo Wrist Score (MWS) [7] were identified as frequently used and valid assessment measurement tools in wrist disorders. However the validation studies most commonly focus specific patient groups or diagnosis (e.g. fractures of the distal radius in the validation of the Patient-Rated Wrist Evaluation Score (PRWE)) and we are still far from a single outcome evaluation system which is reliable, valid and sensitive to clinically relevant change [4–8]. In addition the available self-assessment questionnaires focus on subjective parameters such as pain, usual and specific activities but the range of motion (ROM) as an essential objective parameter in wrist disorders is rarely considered. The Patient-Rated Wrist Evaluation Score (PRWE), for example, presents a wrist specific outcome instrument but it does not depict photographs to allow for a patient-based evaluation of the range of motion. The Disabilities of the Arm, Shoulder and Hand (DASH) score presents a frequently used and established self-assessment score for the general upper limb function but it does not constitute a wrist-specific rating instrument.

Therefore the purpose of this prospective study was to develop and validate an all-purpose Munich Wrist Questionnaire (MWQ) without limitations in the applicability regarding diagnosis or specific disorders for a patient-based follow-up examination considering subjective (pain, work and activities of daily living) as well as objective parameters (range of motion) in a heterogeneous patient collective.

The study protocol was approved by the local ethics committee (Ethics Committee of the medical faculty, Klinikum rechts der Isar, Technical University of Munich, Germany; study number 5316/12).

## Methods

### Development of the scoring system

**Fig. 1:**
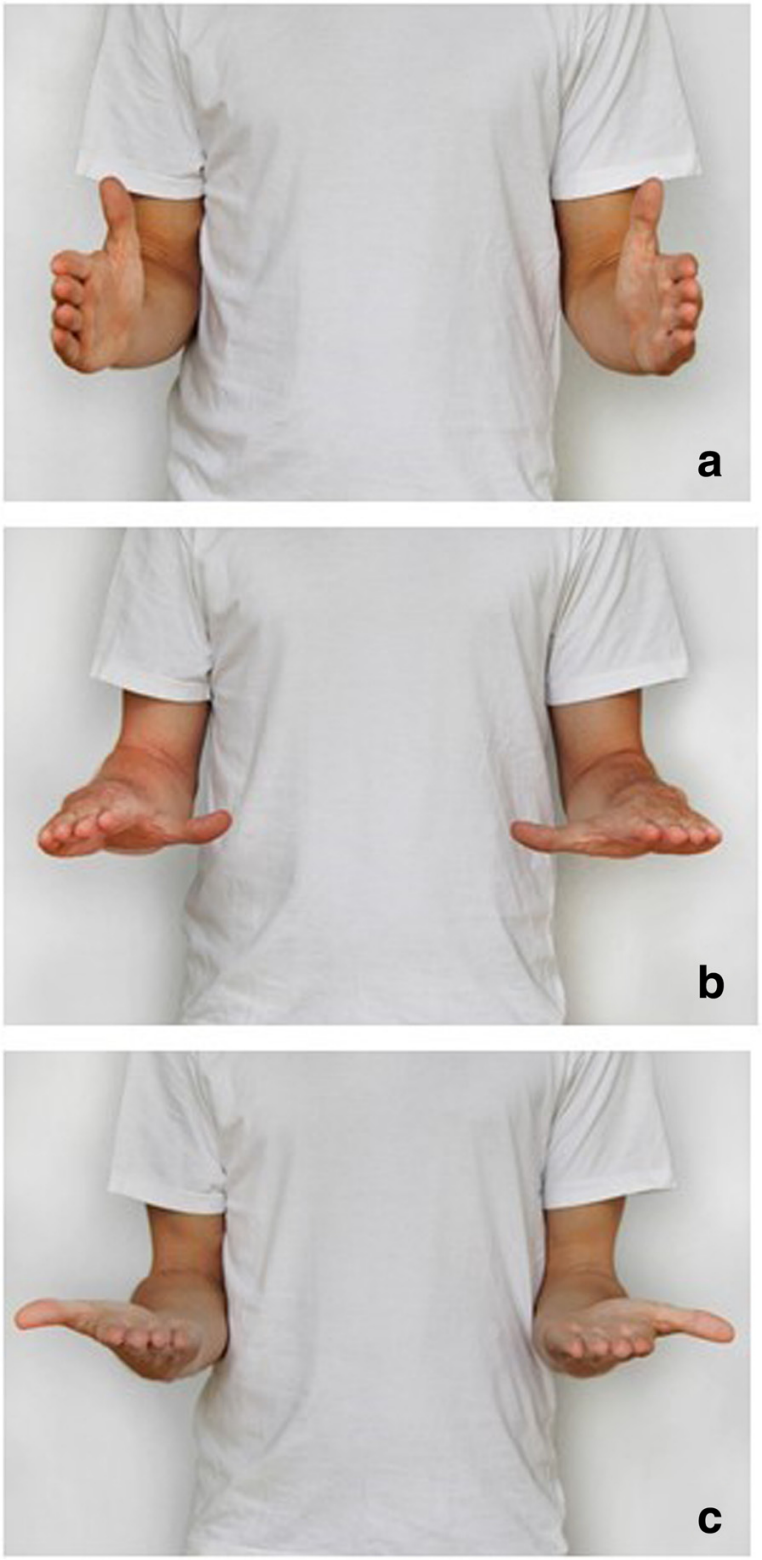
Functional abilities depicted as photographs: pronation/supination. **a** neutral position; **b** pronation; **c** supination

**Fig. 2:**
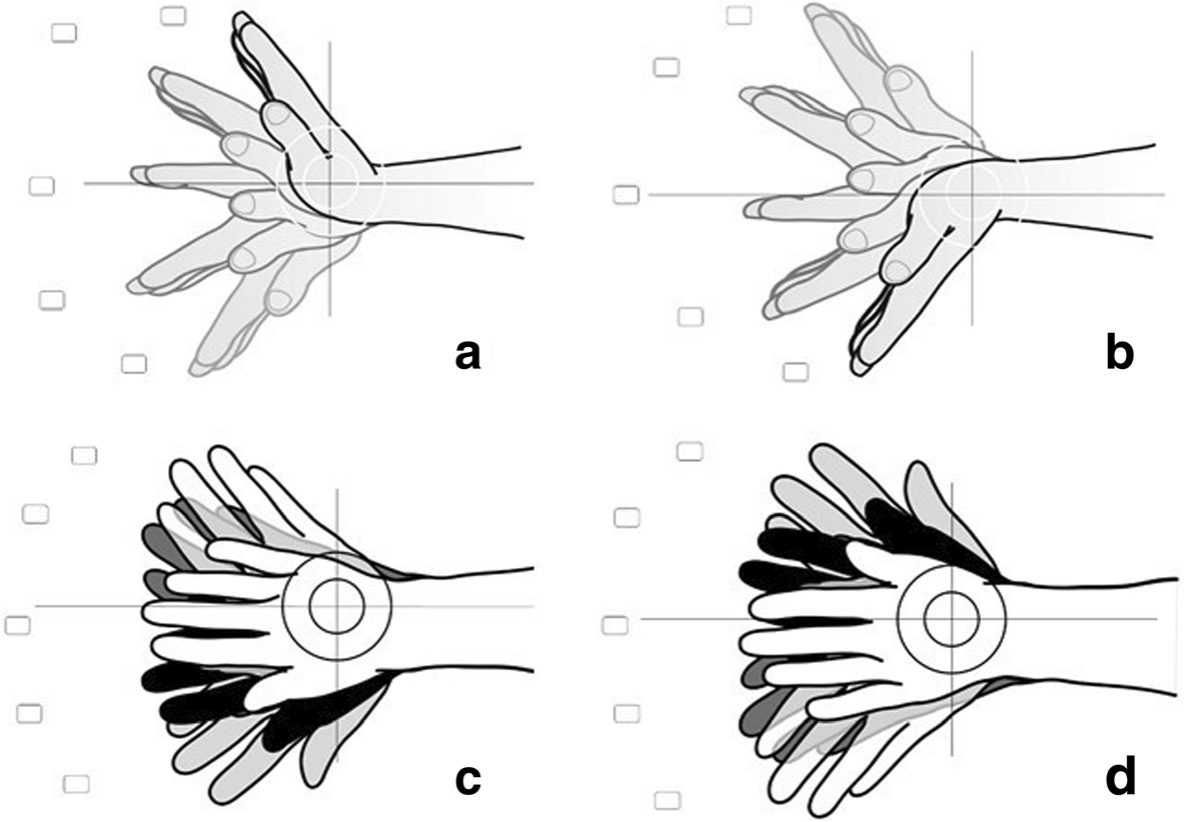
Functional abilities depicted as photographs: flexion/extension and abduction/adduction. **a** extension/flexion of the right hand; **b** extension/flexion of the left hand; **c** abduction/adduction of the right hand; **d** abduction/adduction of the left hand

To capture all aspects of the wrist function each scale of the DASH, the PRWE, the CBS and the MWS was analyzed for items either addressing general topics or specific items. Subsequently a matching of the general topics was performed and the dedicated items underwent a fusion to the final MWQ’s item (Additional file 1). Typical functional abilities were depicted as photographs (see Figs. 1 and 2) to assess the range of motion. Finally the MWQ contains 16 items addressing three domains: pain (five items), work and activities of daily living (work/ADL) (seven items) and wrist function including range of motion and grip strength (four items). The maximum value for all subjective parameters (subscales pain and work/ADL) is 120 out of 250 points (objective parameters (function) 130 out of 250 points) which means a subjective-objective ratio of almost 1:1. The overall score is than converted to a scale of 100 % whereas a value of 100 % indicates an excellent result and a value of zero percent a poor result. The MWQ can be downloaded from our official homepage.

### Patient collective

A cohort of 100 consecutive patients who had suffered from traumatic soft tissue and/or osseous injures as well as degenerative disorders of the wrist joint were asked to complete all questionnaires at the outpatient clinic. All persons gave their informed consent prior to their inclusion in the study. People with limited legal capacity, under legal supervision or suffering from psychiatric diseases, dementia or other cognitive diseases were excluded.

### Testing and evaluation of measurement qualities

#### Floor and ceiling effects

According to McHorney et al. [9] floor and ceiling effects exist, if more than 15 % of the patients achieve the lowest or highest possible score. Similarly we defined the presence of floor or ceiling effects, if more than 15 % of our patient collective would achieve the lowest (0 points) or highest (100 points) possible score of the MWQ.

#### Internal consistency

Internal consistency is defined by the degree of interrelation among the tested items [10]. The subscales are based on a reflective model in which all items are defined by a manifestation of the same underlying construct. According to previous published studies, Cronbach’s alpha was calculated per subscale and a score above 0.70 was considered as sufficient homogeneity of the subscales’ items [11–13].

#### Test-retest reliability

Test-retest reliability is defined as the extent to which scores of the same patients under the same conditions coincide in repeated measurements [10]. The time period between the repeated measurements should be long enough to prevent from recall of the tested items, and moreover should be short enough to ensure that no change of the clinical symptoms has occurred [11]. In this study a time period of 14 to 21 days after the initial examination was chosen to assess test-retest reliability. Intraclass correlation coefficients (ICC) were calculated and positive reliability was assumed when the ICC was at least 0.70 for all tested subscales [11].

#### Construct validity

Construct validity is defined as the degree to which the scores of a self-assessment instrument are consistent with a priori hypothesis, based on the assumption that the instrument validly measures the construct to be measured [10]. Construct validity was assessed by correlating the subscales “pain” and “work/ADL” of the MWQ with the subscales “pain” and “activities” of the PRWE. The subscale “function” of the MWQ was correlated with the subscale “range of motion/grip strength” of the MWS. The Pearson correlation coefficient (PCC) was calculated. Similar to previous studies, a positive construct validity was assumed when the PCC was at least 0.70 for all measured subscales [13, 14].

#### Responsiveness

Responsiveness is defined as the ability of an instrument to detect changes over time of the construct to be measured [10]. Responsiveness was evaluated four to six months after the initial presentation of the patient. To assess responsiveness patients completed the MWQ and a Global Perceived Effect (GPE) score consisting of only one question per subscale on the patients’ subjective opinion regarding improvement or worsening during the last months. A list of potential answers contained seven categories (much better (+3), better (+2), somewhat better (+1), no change (0), somewhat worse (–1), worse (–2), much worse (–3)) for each subscale of the MWQ. The time period of four to six months was chosen to be long enough to allow for a clinical change, and short enough to ensure that the patients are able to recall their health state during their initial presentation. The Spearman’s correlation coefficient (SCC) was calculated. SCC between the change of the MWQ and the GPE score of at least 0.40 was assumed to indicate positive responsiveness [12, 13].

#### Correlation of the MWQ with established wrist scores

We supposed that at least a moderate correlation would be obtained between the new MWQ and already established wrist rating systems (DASH, PRWE, CBS, MWS). The PCC was calculated followed by a linear regression analysis. A positive correlation was assumed when the PCC was at least 0.70.

#### Statistical analysis

The results were compared by calculating the SCC and PCC with a linear regression analysis. A *p*-value <0.05 determined significance.

## Results

### Patients and study design

**Fig. 3:**
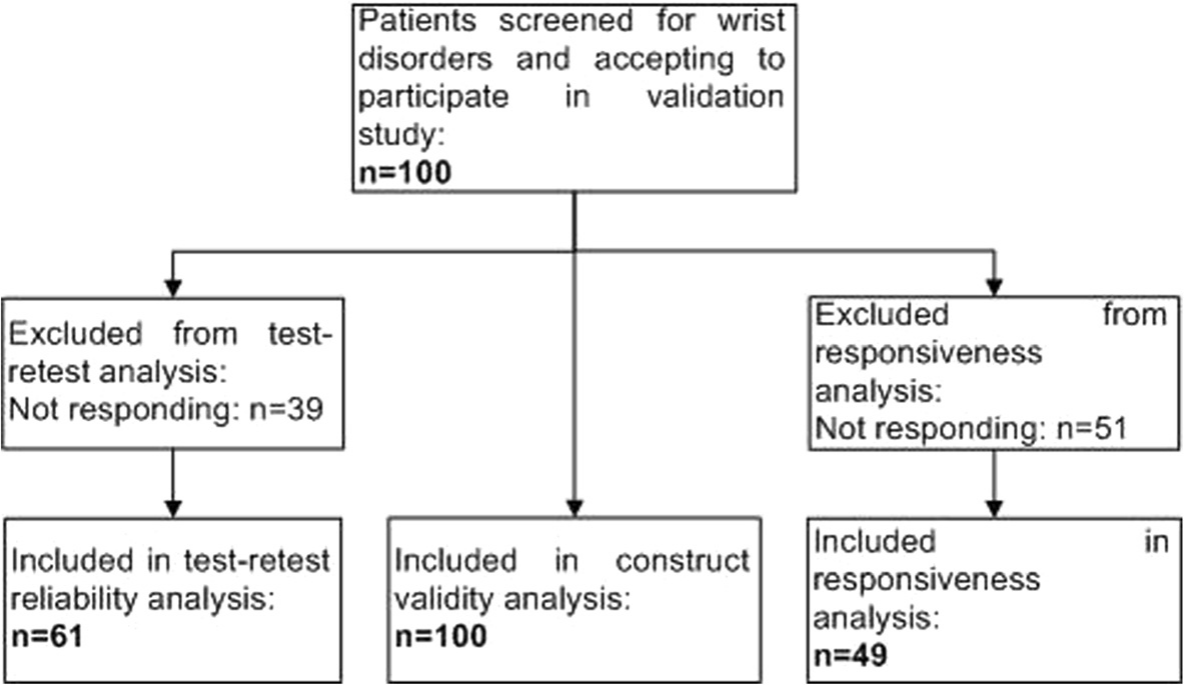
Clinical study profile; flowchart of the study process

**Fig. 4:**
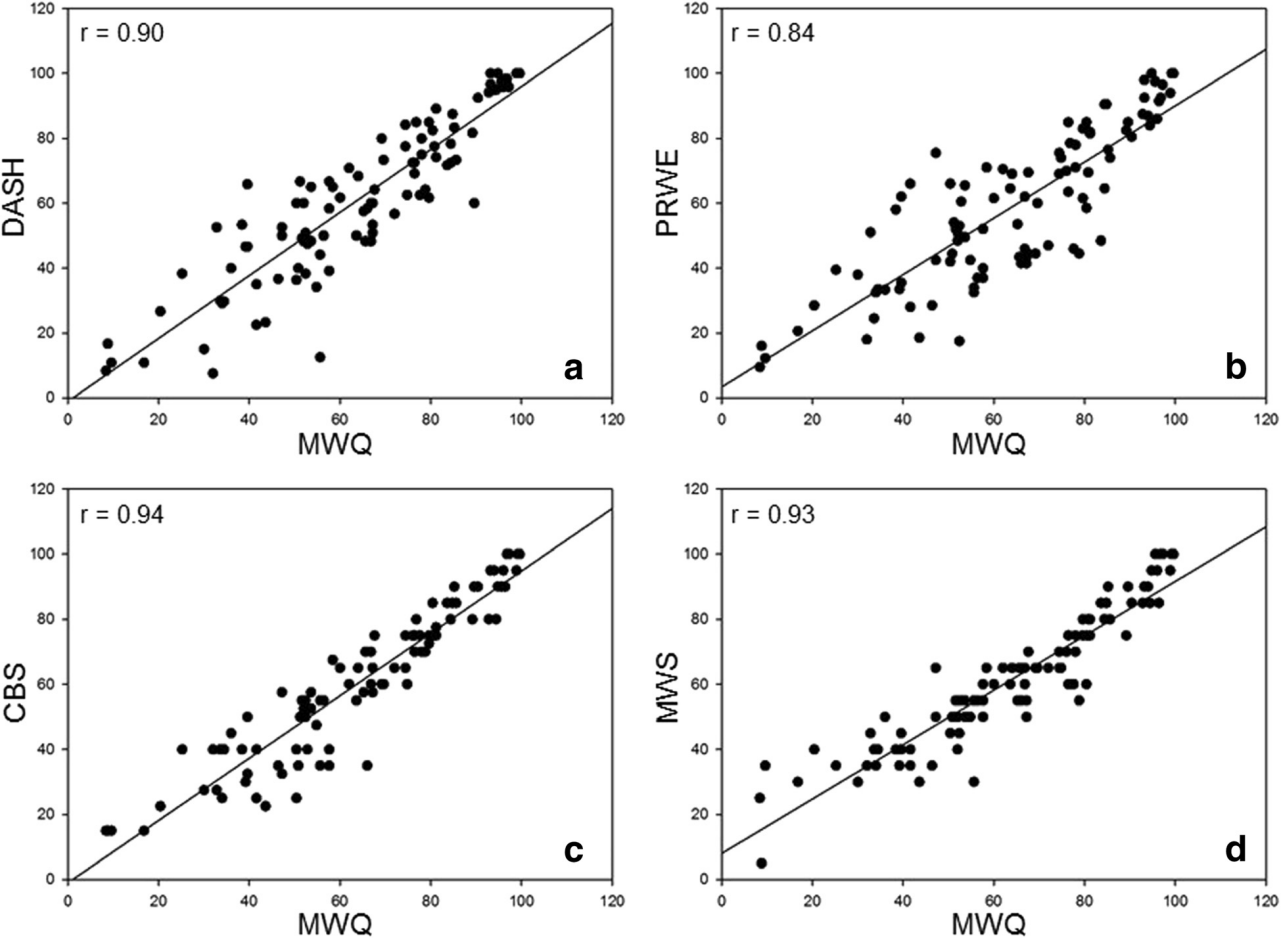
Simple regression scatter plots of the correlation between the MWQ and the DASH (**a**, *n* = 100), the PRWE (**b**, *n* = 100), the CBS (**c**, *n* = 100) and the MWS (**d**, *n* = 100), Solid lines represent the linear regression. Pearson’s correlation coefficients (r) are given in each panel. DASH, Disabilities of the Arm, Shoulder and Hand Score; PRWE, Patient-Rated Wrist Evaluation Score; CBS, Cooney and Bussey Score; MWS, Mayo Wrist Score

Validity, reliability and responsiveness of the MWQ were determined in a prospective, clinical study. Between August 2012 and November 2013 100 consecutive patients (mean age 41 years, SD 16.3 years; range, 18–77 years) were asked to complete the MWQ, the DASH, the PRWE, the CBS and the MWS at initial presentation for evaluating validity. Completion of the MWQ lasted about eight minutes (mean time 7.7 min, SD 2.2 min, Min. 4.0 min, Max. 15.7 min). Table 1 summarizes patient’s diagnosis representing a wide spectrum of traumatic and degenerative wrist disorders. Figure 3 shows the clinical study profile. Figure 4 shows the results of the correlation between the MWQ and frequently used wrist rating systems. The PCC between the MWQ and the DASH was 0.90, 0.84 for the PRWE, 0.94 for the CBS and 0.93 for the MWS (*p* < 0.05).

**Table 1:** Study population, *n* = 100

Diagnosis	Total (*n* = 100)	Men (*n* = 49)	Women (*n* = 51)
Distal radius fracture	35	13	22
Metacarpal fracture	15	9	6
Scaphoid fracture	8	8	0
Other carpal fractures	5	4	1
TFCC tear	12	4	8
Synovitis	8	2	6
SL ligament tear	6	4	2
Wrist OA	6	4	2
Traumatic nerve injury	3	1	2
Wrist contusion	2	0	2

*TFCC* triangular fibrocartilage complex, *SL* scapho-lunate, *OA* osteoarthritis

### Floor and ceiling effects

None of the patients achieved the lowest possible score but one patient achieved the best score of the MWQ (100 points). Thus there were no floor or ceiling effects to be described.

### Internal consistency

Cronbach’s alpha was calculated for each subscale of the MWQ. Values of at least 0.75 showed a high consistency for all items in one subscale (Table 2).

**Table 2:** Internal consistency (*n* = 100) and test-retest-reliability (*n* = 61)

	Test mean (SD)	Retest mean (SD)	ICC	Cronbach’s α
MWQ total	60.0 (22.1)	65.3 (20.3)	0.82	
Pain	6.1 (1.9)	6.5 (2.1)	0.75	0.82
Work/ADL	5.3 (2.7)	6.3 (2.8)	0.83	0.93
Function	20.7 (8.9)	21.8 (7.4)	0.77	0.75

*SD* Standard Deviation, *ICC* Intraclass Correlation Coefficient, *ADL* Activities of Daily Living

### Test-retest reliability

Retest was performed at a mean of 20 days (SD 16.7 days; range 2–107 days) after the patients’ initial consultation. 61 patients (61 %) returned the completed questionnaire (Fig. 3). Intraclass correlation coefficients (ICC) were between 0.75 and 0.83 for all subscales of the MWQ (Table 2).

### Construct validity

Assessment of construct validity contained a correlation of the subscales of the MWQ with the subscales of the PRWE and of the MWS. PCC of at least 0.86 were calculated for all subscales (Table 3).

**Table 3:** Pearson’s correlation coefficients (r) determined when comparing the subscales of the MWQ to the subscales of the PRWE and the MWS, *n* = 100

MWQ	PRWE (pain)	PRWE (activities)	MWS (function)
Pain	–0.87		
Work/ADL		–0.87	
Function			0.86

*PRWE* Patient-Rated Wrist Evaluation Score, *MWS* Mayo Wrist Score, *ADL* Activities of Daily Living

### Responsiveness

Forty nine patients (49 %) returned the completed MWQ and GPE score 180 days (SD 47.9 days; range 83–291 days) after the initial assessment (Fig. 3). The SCC was 0.61 for pain, 0.65 for work/ADL and 0.64 for wrist related function.

## Discussion

In the present study the development and validation of a new self-assessment score in wrist disorders - the MWQ - is described. Based on a single 16-items tool this questionnaire records subjective as well as objective parameters. With special regard to well-established wrist rating systems (DASH, PRWE, CBS, MWS) a high correlation was found (*p* < 0.05).

### Scientific assessment of outcomes by self-assessment questionnaires

Self-assessment measurement tools in addition to the physician based objective evaluation allow for a comprehensive evaluation of the clinical state. Due to their advantages in financial and logistic concerns [15] standardized outcome assessment of large patient collectives is simplified. Furthermore, avoiding face-to-face contact with the patients eliminates a certain observer bias in terms of the interviewer knowing the purpose of the study. Otherwise self-assessment scores also offer possible sources of bias in terms of incomplete and non-response [16]. In the present study a non-responding rate of 39 % in assessing test-retest reliability and 51 % in responsiveness was found. This is favourably comparable to dropout rates of other validation studies in the current literature [8, 17]. Reminding the participating patients by mail or telephone may constitute sufficient measures to increase the responding rate in future validation studies [16].

### Patients and study design

The presented study collective consisted of 100 consecutive patients with a mean age of 41 years with a female-male ratio of almost 1:1 comparable to other validation studies concerning number of patients, age and gender [5, 12, 15]. The traumatic osseous and ligament injuries, acute inflammatory as well as degenerative diseases of the presented patient collective represent the wide spectrum of wrist disorders (see Table 1). In elbow disorders, several authors prefer such a heterogeneous collective of patients combining different clinical entities for validation of measurement instruments in order to allow for an universal application [18–20]. In the presented study the percentage of traumatic and degenerative disorders remained equal despite of the limited responding rate in the evaluation of test-retest reliability and responsiveness. Therefore the broad application of the MWQ is not limited.

### Internal consistency

Cronbach’s α of at least 0.75 resulting for all subscales of the MWQ stands for a high internal consistency. The different items of the same subscale (e.g. wrist pain) seem to measure the same general construct. The highest value of 0.93 found for the subscale work/ADL did not exceed 0.95 that might indicate item redundancy [20].

### Test-retest reliability

ICCs between 0.75 and 0.83 for all subscales of the MWQ indicate a positive test-retest reliability. In the literature an exact time point for the retest assessment is missing but in many cases a time period of 1 or 2 weeks is considered as appropriate [11]. The patients evaluated in this study were instructed to complete and return the second questionnaire after 14–21 days. Nevertheless, two patients returned the score already after two days increasing the risk of recall-bias. One other patient did only return the score 107 days after the initial visit which may increase the possibility of a change of his clinical state.

### Construct validity

To assess construct validity the relationship between the MWQ and the gold standard in the evaluation of wrist disorders has to be reported. However in the literature no gold standard exists and the subscales of the new self-assessment questionnaire are often compared to established health status measures [21]. The decision was made to correlate the subscales of the MWQ with the subscales of a previously reported validated score. For comparison of the subscales “pain” and “work/ADL” the PRWE score was chosen - a well-established valid, reliable and responsive instrument - as reference score. The subscale “function” of the MWQ was correlated with the subscale “range of motion/grip strength” of the MWS. This decision was made because the range of motion which is depicted as photographs in the MWQ is also theoretically queried in the MWS. Pearson’s correlation coefficients of at least 0.86 resulted for all subscales of the MWQ. Compared to other validation studies these results indicate a high construct validity in a self-reported score [13, 22, 23].

### Responsiveness

The correlation between the change in scores of the first and second MWQ completion and the GPE score showed a range from 0.61 to 0.65 for the subscales pain, work/ADL and wrist related function indicating a positive responsiveness. Since the GPE score contains only one single question, subjective clinical change of the wrist function may have been influenced considerably by persisting symptoms although other symptoms changed considerably. This possibly results in a supposed minor responsiveness, requiring a multi-item instrument [24]. Despite of various statistical tools to determine responsiveness the method of choice remains unclear [25]. The determination of the effective size and the standardized response mean in addition to the GPE score may constitute helpful amendments to assess responsiveness in future validation studies [12].

### Substantial comparison to existing scores

Changulani et al. [1] critically analyzed the available outcome measurement tools for the assessment for wrist and hand function. The PRWE score was identified as a very responsive instrument to evaluate patients with distal radius fractures. However the PRWE score does not allow for a self-assessment of the range of motion and it is not validated in the broad spectrum of wrist disorders. Although it was not possible to validate the MWQ in all wrist disorders, the validation study was performed in a wide spectrum of traumatic and degenerative wrist diseases and its applicability is not limited to specific diagnosis. The DASH score presents an universal score to self-assess the upper extremity as a single functional unit. It was designed to measure the physical function and symptoms of multiple joints of the upper extremity but it does not take into account the range of motion and the grip strength of the wrist joint. The MWQ, however, allows besides the self-assessment of the range of motion also for an evaluation of the grip strength in comparison the healthy or uninjured wrist.

## Limitations

The evaluation of test-retest reliability and responsiveness were conducted at the patients’ homes to avoid financial and logistic burden. Therefore an effect of the change in setting on the test results cannot be excluded. Nonetheless we consider this fact as less relevant since the initial assessment in our clinic as well as the second and the third assessment at home were accomplished in self-administration. Furthermore, responsiveness was assessed by correlating a global perceived effect score with the single subscales of the MWQ. Since the GPE score contained only one single question and the subscales of the MWQ contained between five and seven questions, the GPE score could be less reliable than a multi-item instrument [24] resulting in a reduced interpretability of responsiveness.

Another limitation is that the MWQ has only been tested in the German population and a cross-cultural adaption into other languages and determination of its clinimetric properties has to be conducted before it can be used worldwide.

The patient-based assessment of borderline patients such as highly-trained athletes or frail people being in need for care may be complicated resulting in a reduced universal applicability. However due to the vast majority of patients being potentially evaluated by this tool these drawbacks might be negligibly.

## Conclusions

The MWQ is a self-administrated, valid and reliable tool to assess the most important aspects of the wrist function. Based on the present data the MWQ allows for a qualitative self-assessment of subjective as well as objective parameters (e.g. range of motion) of the wrist joint. The implementation of the MWQ is not restricted to specific wrist disorders or patient groups with the aim of a universal clinical applicability.

### Ethics approval

The study protocol was approved by the local ethics committee (Ethics Committee of the medical faculty, Klinikum rechts der Isar, Technical University of Munich, Germany; study number 5316/12).

### Availability of data and materials

All data concerning the validation of the MWQ is contained within the manuscript. Further study regarding the applicability of the MWQ will be published soon.

## Additional file

Additional file 1: Munich Wrist Questionnaire. (PDF 976 kb)

## Abbreviations

ADL: activities of daily living
CBS: cooney and bussey score
DASH: disabilities of the arm, shoulder and hand score
GPE: global perceived effect
ICC: intraclass correlation coefficient
MWQ: munich wrist questionnaire
MWS: mayo wrist score
PCC: pearson’s correlation coefficient
PRWE: patient-rated wrist evaluation score
ROM: range of motion
SCC: spearman’s correlation coefficient
