# Supplementary material for: The Munich Wrist Questionnaire (MWQ) – development and validation of a new patient-reported outcome measurement tool for wrist disorders
Source: BMC Musculoskelet Disord. 2016 Apr 14;17:167. doi: 10.1186/s12891-016-1029-4 (PMC4832466; doi:10.1186/s12891-016-1029-4)
Supplement: Additional file 1: — Munich Wrist Questionnaire. (PDF 976 kb) [file 12891_2016_1029_MOESM1_ESM.pdf]

Munich Wrist Questionnaire

Please answer **every question** with exactly **one cross!**

Date:

Sex: ☐ male ☐ female

Affected wrist: ☐ right ☐ left ☐ both wrists

Working ☐ yes, as: ____________________

☐ no, if no, because of the wrist? ☐ yes ☐ no

Pensioner ☐

Housewife ☐

Do you take drugs because of pain in the affected wrist?

☐ yes, if yes which drugs ____________________

____

☐ no ____________________

right-handed ☐ left-handed ☐

For which wrist do you fulfill the questionnaire?

☐ right ☐ left

Pain

1. How severe is your pain at rest?

none — little — moderate — strong — intolerable

☐ ☐ ☐ ☐ ☐ ☐ ☐ ☐ ☐ ☐ ☐

2. How severe is your pain during acitivities of daily living?

none — little — moderate — strong — intolerable

☐ ☐ ☐ ☐ ☐ ☐ ☐ ☐ ☐ ☐ ☐

3. How severe is your pain during carrying a filled shopping bag or a briefcase?

none — little — moderate — strong — intolerable

☐ ☐ ☐ ☐ ☐ ☐ ☐ ☐ ☐ ☐ ☐

4. How often do you have pain in the treated wrist?

never — rare — sometimes — often — always

☐ ☐ ☐ ☐ ☐ ☐ ☐ ☐ ☐ ☐ ☐

5. How satisfied are you with the treatment result?

excellent — moderate — not at all

☐ ☐ ☐ ☐ ☐ ☐ ☐ ☐ ☐ ☐ ☐

Work and activities of daily living

6. Do you have difficulties when opening a closed glass of honey or marmalade?

none — little — moderate — strong — impossible

☐ ☐ ☐ ☐ ☐ ☐ ☐ ☐ ☐ ☐ ☐

7. Do you have difficulties when turning a key in the keyhole?

none — little — moderate — strong — impossible

☐ ☐ ☐ ☐ ☐ ☐ ☐ ☐ ☐ ☐ ☐

8. Do you have difficulties when using a knife to prepare a meal?

none — little — moderate — strong — impossible

☐ ☐ ☐ ☐ ☐ ☐ ☐ ☐ ☐ ☐ ☐

9. Do you have difficulties when pressing a door handle?

none | little | moderate | strong | impossible

10. Do you have difficulties when writing on a keypad or playing the piano?

none | little | moderate | strong | impossible

11. Do you have difficulties when brushing your teeth?

none | little | moderate | strong | impossible

12. Could you return to your former work after treatment?

Former work possible

Limited in former work due to the treated wrist

Able to work, but unemployed

Not able to work due to the treated wrist

Grip strength

13. How do you assess the grip strength of the treated wrist in comparison to your uninjured wrist? Grip strength corresponds to

100% | 75% | 50% | 25% | 0%

Range of motion

14. Pronation / Supination

Is it possible for you to reach the shown positions with your right and left hand? Please note that your elbows are closed to your body!

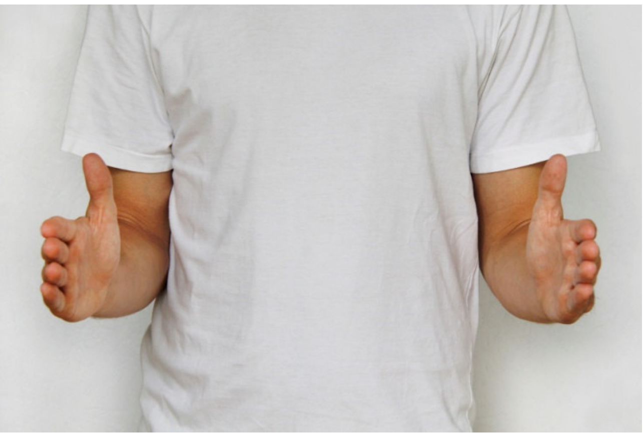

Right hand:

- ☐ yes
- ☐ no

Left hand:

- ☐ yes
- ☐ no

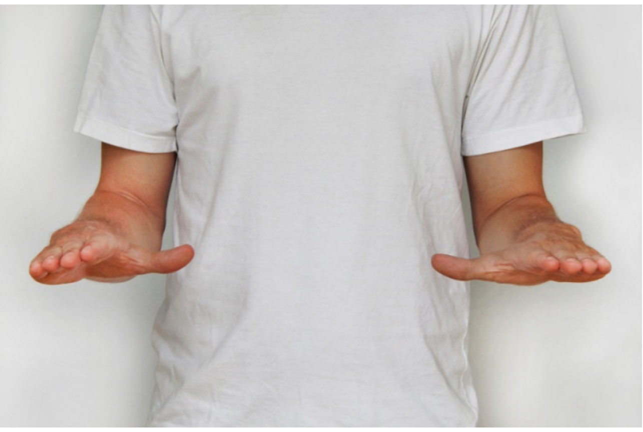

Right hand:

- ☐ yes
- ☐ no

Left hand:

- ☐ yes
- ☐ no

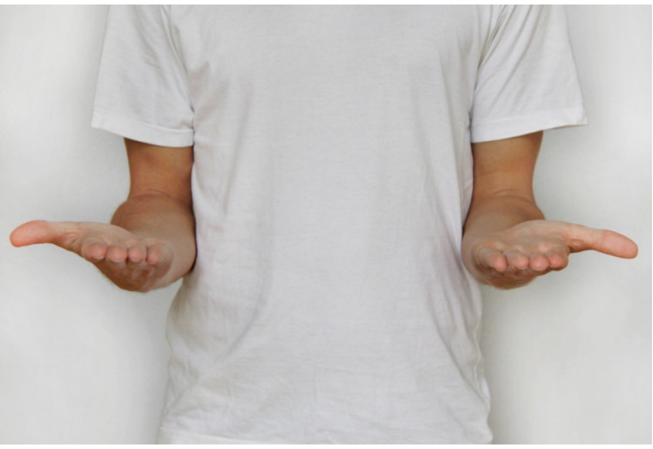

Right hand:

- ☐ yes
- ☐ no

Left hand:

- ☐ yes
- ☐ no

15. Please use the attached illustrations / figures to answer the following two questions (figure 1, 2, 3 and 4):

1. Dorsal extension / palmar flexion (figure 1 and 2)

Please put your right and left hand on the attached figures 1 and 2:

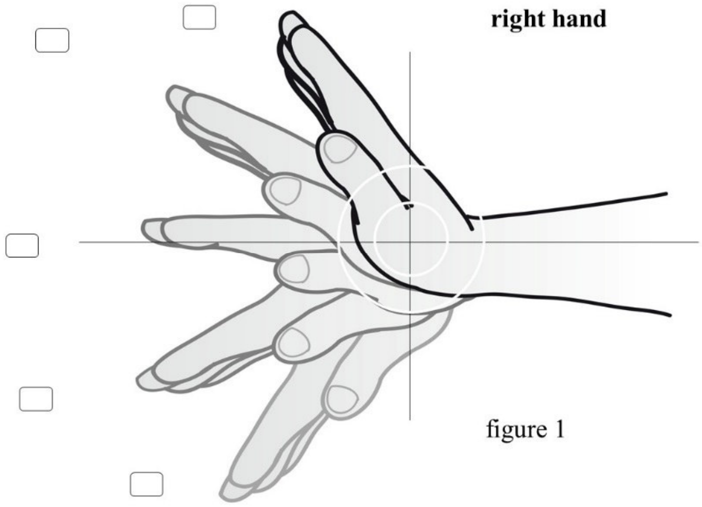

figure 1

The black circle corresponds to the rotation center of the wrist. Now move your hand according to the illustration and check which positions can be reached. Please note that your forearm should **not** change the position. The movement should only take place in your wrist!

If you can only reach the starting position, please make a cross at the starting position.

2. Radial abduction / ulnar abduction (figure 3 and 4)

Please put your right and left hand on the attached figures 3 and 4:

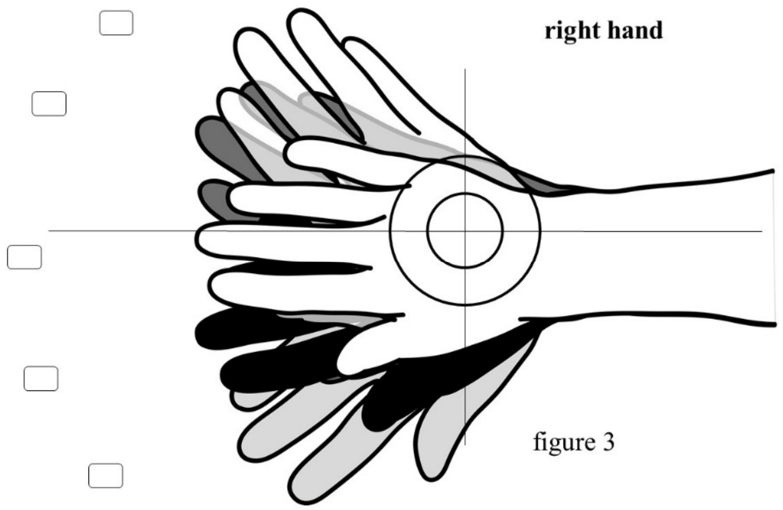

figure 3

The black circle corresponds to the rotation center of the wrist. Now move your hand according to the illustration and check which positions can be reached. Please note that your forearm should **not** change the position. The movement should only take place in your wrist!

If you can only reach the starting position, please make a cross at the starting position.

Thank you very much for your efforts!

right hand

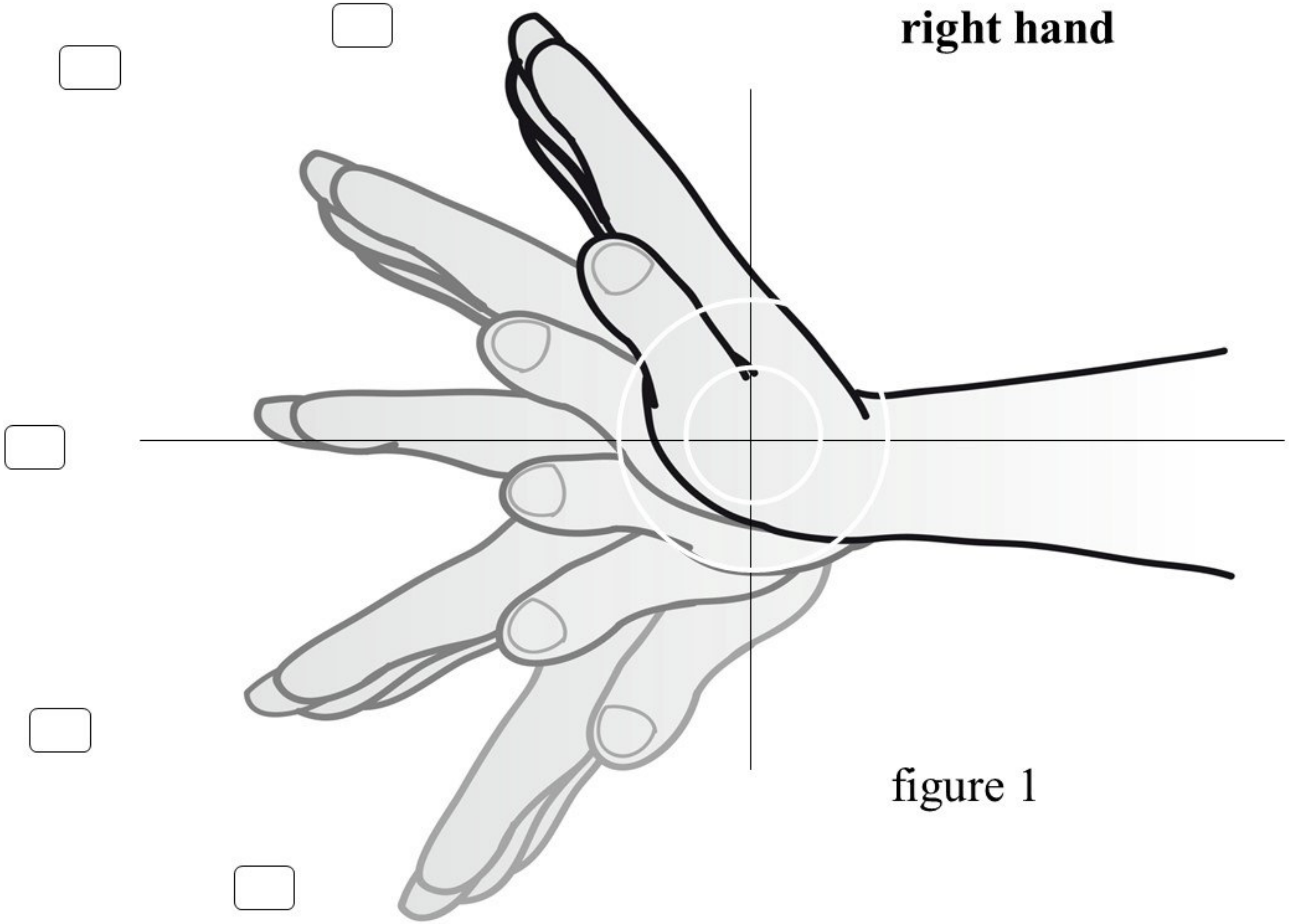

figure 1

left hand

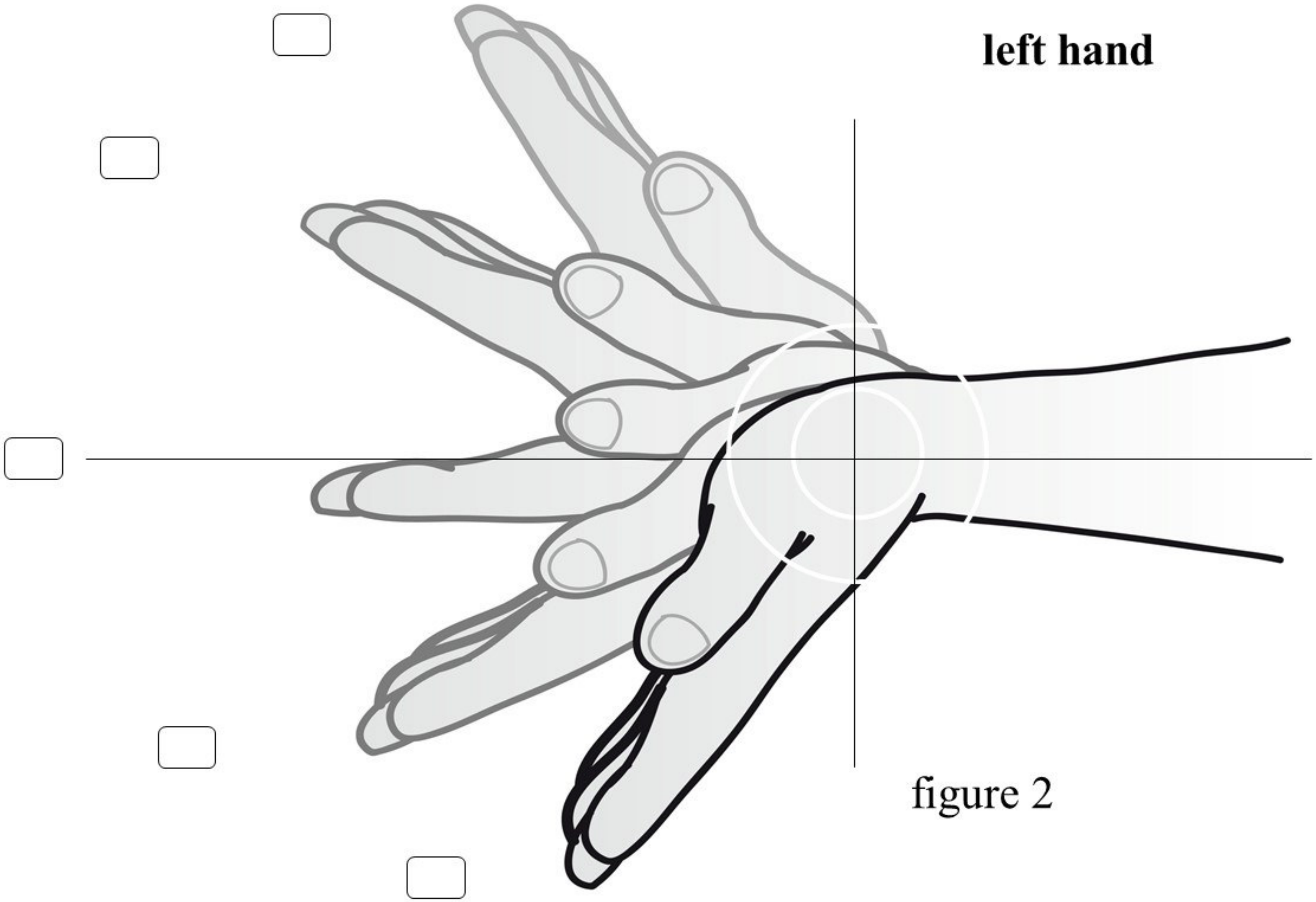

figure 2

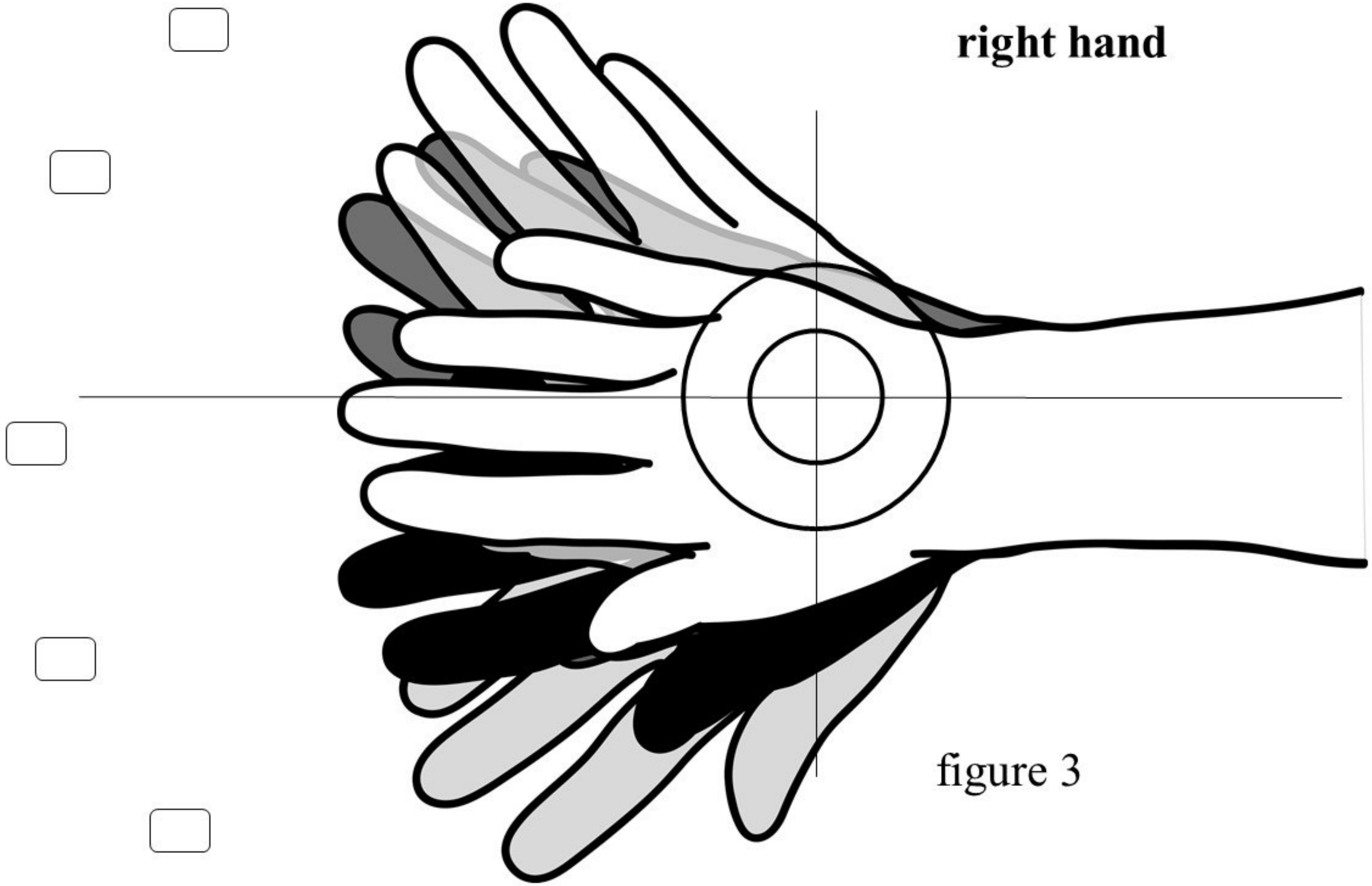

figure 3

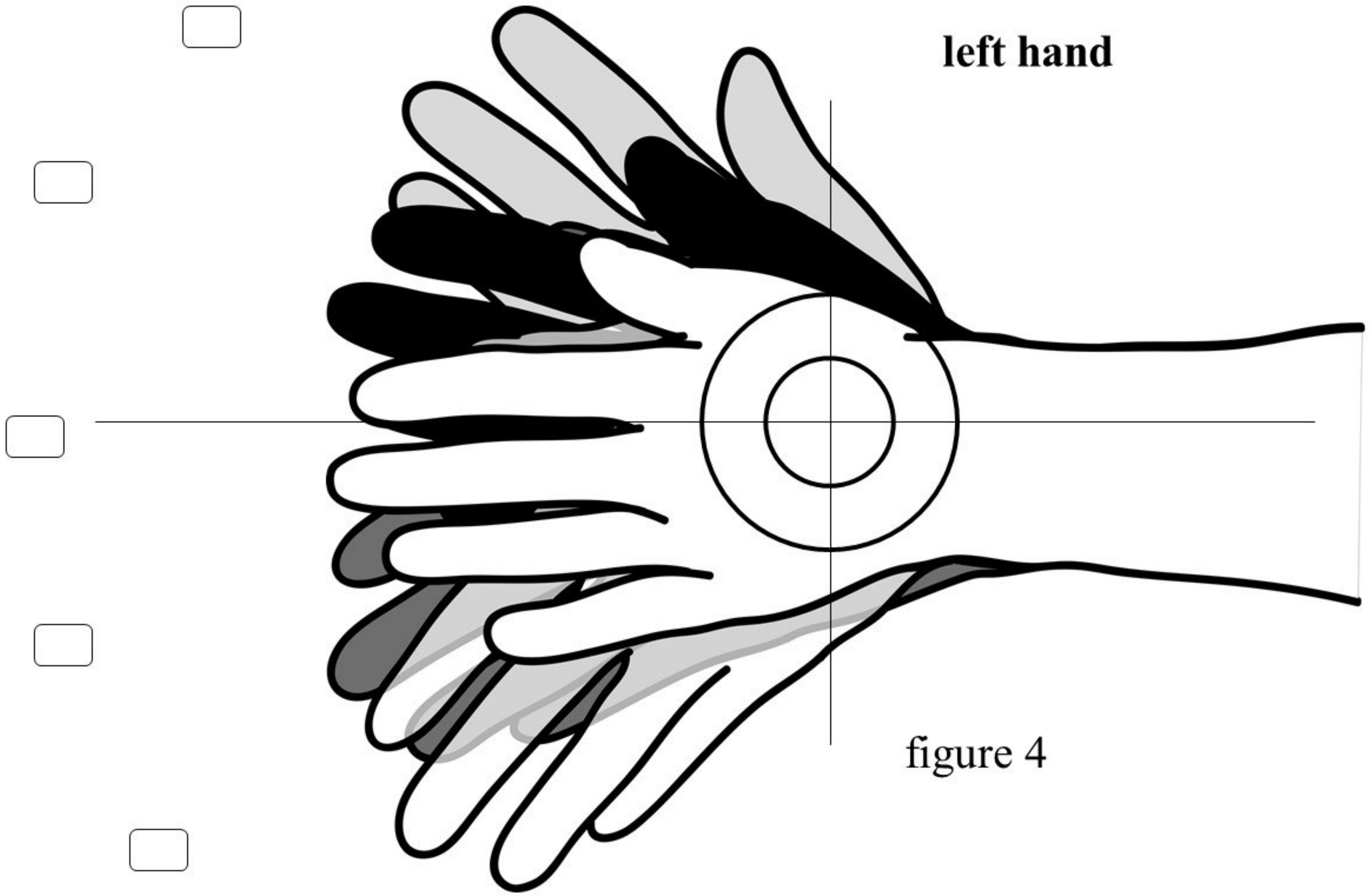

figure 4
